# Hybrid Deep Learning Neural Networks for Small-Molecule Organic Amine Gas Recognition and Prediction Based on MOS Sensor Array

**DOI:** 10.3390/s26196012

**Published:** 2026-09-23

**Authors:** Xinglei Zhao, Chenjun Ning, Wenqi Fan, Chen Chen, Shanshan Li, Jiale Zheng, Lei Li

**Affiliations:** 1 State Key Laboratory of Petroleum Pollution Control, Beijing 102206, China; 13718649673@163.com (X.Z.); 18811392699@163.com (C.N.); roymaii@foxmail.com (J.Z.); 2 CNPC Research Institute of Safety and Environmental Technology, Beijing 102206, China; 3 College of Instrumentation & Electrical Engineering, Jilin University, Changchun 130021, China; cchen@jlu.edu.cn; 4 Key Laboratory of Geophysical Exploration Equipment, Ministry of Education of China, Jilin University, Changchun 130021, China; 5 Department of Electronics and Electrical Engineering, Changchun University of Technology, Changchun 130012, China; 2202404094@stu.ccut.edu.cn

**Keywords:** MOS sensor array, deep learning, small-molecule organic amines, concentration prediction, Bi-GRU

## Abstract

Carbon capture, utilization and storage (CCUS) is a critical technology for the fossil energy industry to achieve the dual carbon goals. Organic amine-based absorption methods, represented by mono-ethanolamine (MEA), methyl-diethanolamine (MDEA) and 2-amino-2-methyl-1-propanol (AMP), are currently the most widely adopted approaches for carbon dioxide capture. If the concentration of leaked organic amines exceeds the safety threshold, inhalation will cause severe respiratory irritation and even serious illnesses in humans. Accordingly, in situ monitoring of organic amine concentrations in waste gas is of great significance for process optimization, environmental protection, early health warning, energy conservation and emission reduction. In this study, a metal oxide semiconductor (MOS) sensor array was developed to identify categories and concentration variations in small-molecule organic amines. To realize effective gas classification and accurate concentration prediction, single-component gas data and mixed gas data with varying concentrations of the three amines collected in the laboratory were adopted to train a one-dimensional convolutional neural network (1D-CNN) and a bidirectional gated recurrent unit (Bi-GRU) via five-fold cross-validation. The proposed model achieved a classification accuracy of 0.9969 ± 0.0006. For MEA concentration prediction, the optimal determination coefficient (R^2^), root mean square error (RMSE), and mean absolute error (MAE) were 0.9969, 2.1306 and 1.5503, respectively. The experimental results demonstrate that the proposed method possesses promising practical application prospects in in situ gas monitoring and early hazard warning.

## 1. Introduction

Carbon capture, utilization and storage (CCUS) is a vital technology for fossil energy industries to achieve dual carbon goals, adopting alkylamine-based chemical absorption for selective CO_2_ removal [1]. A downstream water washing system serves as a key emission reduction unit, recovering entrained amine vapors, droplets and partial degradation products to mitigate amine emissions [2]. However, washed waste gas still contains residual organic amines in gaseous, droplet and aerosol states. Water washing effectively removes mono-ethanolamine (MEA) but fails to eliminate trimethylamine droplets, ammonia and aldehyde by-products and cannot control aerosol-state amine pollutants efficiently [3,4,5]. These trace residual amines trigger air pollution and threaten ecological and human health.

Traditional methods for organic amine detection mainly rely on laboratory-based analytical techniques, among which gas chromatography-mass spectrometry (GC-MS) is one of the most widely used and authoritative approaches. GC-MS combines the high separation capability of gas chromatography (GC) with the strong qualitative and quantitative power of mass spectrometry (MS), enabling precise identification and quantification of complex volatile organic compound mixtures [6]. In addition, high-performance liquid chromatography (HPLC), ion chromatography (IC), spectrophotometry, and potentiometric titration are also commonly employed for organic amine analysis, each suitable for amine compounds with different polarities, molecular weights, and existing forms. Although traditional instrumental methods such as GC-MS are unparalleled in accuracy and sensitivity, providing solid foundational data for scientific research on organic amines, they exhibit significant shortcomings in terms of real-time, on-site, and portable applications. These inherent limitations have driven an urgent need for new, miniaturized, and intelligent gas sensing technologies.

To overcome the limitations of traditional analytical methods, researchers have turned their attention to gas detection technologies based on chemical sensors. Gas sensors relying on principles such as metal oxide semiconductors (MOS), electrochemistry, and optics have demonstrated great application potential in fields including environmental monitoring, industrial process control, food safety, and medical diagnostics, owing to their advantages of small size, low cost, low power consumption, and fast response. Researchers have developed various sensors based on different principles, among which metal oxide semiconductor (MOS) sensors have been the most extensively and thoroughly studied [7,8,9]. However, when conventional single gas sensors are used for the detection of complex gas mixtures, they suffer from a serious issue of cross-sensitivity—namely, the sensors exhibit similar responses to different gases, making it difficult to distinguish target analytes with similar structures and chemical properties, such as MEA and MDEA [10]. To address this challenge, sensor arrays have emerged as a solution.

The core idea of the sensor array is that it no longer relies on a single sensor with perfect selectivity, but instead uses an array of multiple non-specific sensors. Each sensor in the array has a broad response range, but its sensitivity and response pattern to different gases vary. When one or more gases flow across the sensor array, each sensor produces a response signal, and the combination of all sensor response signals forms a high-dimensional response vector, which can be regarded as a “digital fingerprint” or “smell print” of the gas mixture under test. Since different gas mixtures produce distinct and reproducible fingerprints, the type and composition of the gases can be identified by comparing these fingerprints [11,12]. After obtaining the multidimensional response data from the sensor array, advanced pattern recognition algorithms need to be applied to analyze these data and extract useful classification or quantitative information from them. This strategy of “sensor array + pattern recognition” transforms the gas identification problem from a physical or chemical selectivity issue at the sensor level into a pattern classification problem at the mathematical and computational level, offering a highly promising approach for distinguishing complex gas mixtures with similar structures and varying concentrations. In the field of gas sensing, the introduction of machine learning marks a shift in research direction and paradigm. For organic amine molecules with similar structures, such as MEA and MDEA, traditional methods struggle to distinguish them, whereas machine learning models can learn to discriminate them by analyzing the subtle differences in the entire response vector. Among the numerous machine learning algorithms, Support Vector Machine (SVM) [13,14], Random Forest (RF) [15], and Logistic Regression (LR) are the most widely applied and robust classical models. Although classical machine learning algorithms have achieved great success in gas sensing tasks, they usually rely on handcrafted feature extraction steps. This process is not only cumbersome, but its effectiveness also heavily depends on the experience and knowledge of experts. For complex and unstructured sensor response data, handcrafted features often fail to capture the most essential and relevant information.

Deep Learning (DL) offers an ultimate solution to this problem. The core advantage of deep learning lies in its “representation learning ability”, which enables it to automatically and hierarchically learn and extract features directly from raw data (such as images and time-series signals) without any human intervention. This characteristic enables it to demonstrate stronger capability and higher efficiency than traditional machine learning methods when dealing with high-dimensional and complex sensor signals. A study employed a 1D-CNN to process raw experimental sample data of methane, carbon monoxide, ethylene, and their mixtures, achieving a recognition accuracy as high as 99.98%, demonstrating the excellent performance of 1D-CNN in handling raw gas sensor data [16]. In addition to classification tasks, 1D-CNN has also been successfully applied to the quantitative inversion of gas concentrations, showcasing the great potential of deep learning in improving the quantitative accuracy of sensors [17]. For a typical time-series process such as gas sensing, the current sensor response depends not only on the current gas concentration but also on historical concentration values, meaning that the adsorption–desorption process of gas molecules on the sensor surface exhibits dynamic and memory characteristics. Recurrent Neural Networks (RNNs) are precisely designed to handle such sequential data, in which Long Short-Term Memory (LSTM) and Gated Recurrent Unit (GRU) were proposed. A study proposed a hybrid CNN-RNN architecture for real-time prediction of gas concentrations in mixtures of ethylene, carbon monoxide, and methane. In this model, the CNN part first extracts spatial features from the multi-dimensional sensor response data, and these features are then fed into an LSTM network for temporal modeling, ultimately producing point-by-point predictions for each gas concentration. Experimental results show that when predicting the mixed gas concentrations of carbon monoxide and ethylene, the model achieved root mean square errors (RMSE) as low as 35.11 ppm and 1.33 ppm, respectively, outperforming several comparative models [18]. Another study utilized an LSTM network to process time-series data collected by an array of eight metal oxide semiconductor sensors for distinguishing nitrogen dioxide (NO_2_), formaldehyde (HCHO), and their mixtures. By learning the dynamic differences in adsorption/desorption processes of different gases, the model achieved a classification accuracy as high as 92%, demonstrating the effectiveness of RNNs in handling dynamic and distinguishable gas response processes [19].

Above all, in this study, we developed a commercial MOS sensor array and established dynamic features and quantile transformation to identify MEA, MDEA, and AMP. Single-component gas data and mixed gas data with varying concentrations of three amines collected in the laboratory are used to train a one-dimensional convolutional neural network (1D-CNN) and a bidirectional gated recurrent unit (Bi-GRU) via five-fold cross-validation. The research results can guide the design optimization and operational regulation of water washing systems. Furthermore, the integration of monitoring and control systems is promoted to achieve intelligent feedback and optimized control based on in-site emission data. Relevant operating parameters can be dynamically adjusted to ensure compliant emissions while reducing operational costs.

## 2. Experimental Section

### 2.1. Sensors Array

Eight chemiresistive metal oxide semiconductor (MOS) sensors were selected to assemble the sensor array, including two TGS2600, one TGS2602, two TGS822, one MQ3, one MQ135 and one MP503. Among them, TGS822, TGS2602 and TGS2600 were purchased from Figaro Engineering Inc., Shanghai, China, while MQ3, MQ135 and MP503 were supplied by Winsen Electronics Co., Ltd, Zhengzhou, China. The performance specifications and optimal operating current of each sensor are presented in Table 1. The target gases and detection range in Table 1 are officially indicated in the sensor manuals, but in the experimental process, it was found that these sensors can be used at lower concentrations and for the detection of MEA, MDEA and AMP, utilizing the cross-sensitivity characteristic of MOS sensors. The selection of these sensors was based on known sensitivity to amines, complementary cross-sensitivity to ammonia and VOCs, commercial availability, low cost, response intensity, and capability for temperature modulation. In the actual testing process, these sensors met the experimental requirements.

### 2.2. Experimental Platform

The experimental platform adopted the CGS-8 intelligent gas sensing analysis system, which was purchased from Beijing Elite Technology Company, Beijing, China. The overall device is shown in Figure 1. The CGS-8 system consists of a 20 L gas chamber with acrylic material, a gas chamber base and a main control unit. It is equipped with eight independent sensor channels, enabling separate gas testing and operating temperature regulation for each sensor. The gas chamber base integrates an evaporation unit, a fan and a temperature-humidity sensor, as detailed in the inset of Figure 1.

To ensure consistency between model training and testing, all algorithms were implemented in PyCharm (version 2017.1) with Python 3.7.1. The operating system was Windows 11 (×64). The hardware configuration included an Intel Core i9–12900H CPU, Intel Iris Xe Graphics GPU and 16 GB RAM.

### 2.3. Gas Sensing Procedure

The static gas dilution method was applied in all gas tests. All concentrations of target vapors of reagents in the gas chamber were calculated in accordance with Equation (1)

(1) $$Q=\frac{V\;\times \;C\times \;M}{22.4\;\times \;d\;\times \;\rho }\;\times \;10^{-9}\;\times \;\frac{273+T_{R}}{273+T_{B}}$$

where Q is the volume of the injected reagent (mL), V is the volume of the gas chamber (mL), M is the molecular weight (g/mol), C is the concentration of the target vapors (ppm), *d* is the reagent density (g/cm^3^), *ρ* is the reagent purity (%), T_R_ is the temperature of an ideal gas under standard conditions (°C), and T_B_ is the temperature inside the gas chamber (°C).

The specific details are as follows: specific volumes of 99% MEA, 98% MDEA and 98% AMP reagents were drawn out with a microsyringe and injected into three wide-mouth brown glass sampling bottles labeled with MEA, MDEA and AMP. In order to obtain low-concentration target gas (C), all reagents were first diluted to 10% of their original purity with deionized water. Afterward, the required volumes of diluted reagents were stirred thoroughly for 3 min and prepared for gas testing. The same procedure was used for the mixture of MEA, MDEA and AMP.

Prior to formal tests, the sensor array was assembled according to the preset layout. It was assumed that the target gas was uniformly distributed in the gas chamber; the positions of sensors have a relatively small impact on the sensing results, and thus tests for different sensor layouts were not conducted. After installing all sensors, the optimal operating temperatures were set for each sensor as listed in Table 1. In general, the sensors were preheated for 0.5 to 1 h until the baseline became stable. The gas chamber was closed first, then different volumes of diluted reagent were injected into the evaporating dish, which can be seen in Figure 1b inset, through the injection port using a 10 µL microsyringe, one by one. Meanwhile, the evaporator and the fan on the test platform were turned on to ensure even distribution of vaporized gas inside the chamber. After the sensor responses stabilized, the gas chamber was lifted, and room air was allowed to enter the chamber, followed by switching off the evaporator and fan. When the response curve intensity recovered to the baseline, single gas concentration testing was completed. For single-concentration testing, the response curve was divided into three phases: response phase, steady phase and recovery phase. The response phase refers to the duration from when the sensor contacts the target gas until its response reaches 90% of the steady-state value. The steady phase follows the response phase, during which the sensor output remains relatively stable, and the response value has a definite correlation with gas concentration. The recovery phase starts after opening the gas chamber, lasting until the sensor response drops to approximately 5% of its baseline value. Repeating this procedure, when the testing of all target gas concentrations was finished, the measured resistance values of sensors and response values (R_a_/R_g_) were saved into CSV files for subsequent model input [20]. The concentrations of each of the three target gases ranged from 10 ppm to 100 ppm, and the corresponding dynamic response curves of MP503 sensors are presented in Figure 2. The dynamic response curves of 7 other sensors for the three target gases and ternary mixtures are shown in Figures S1 and S2 in the Supplementary Materials. The dynamic response testing of the three target gases for each sensor was repeated three times. The sampling interval was set to 1 s. The sensor response was defined as R_a_/R_g_, where R_a_ represents the sensor resistance in air, and R_g_ denotes the sensor resistance in the target gas. For the mixed target gases, the detailed concentration combinations of the three target gases are listed in Table 2.

## 3. Software Algorithm

### 3.1. 1D CNN Module

The basic architecture of a one-dimensional convolutional neural network (1D CNN) is similar to that of a two-dimensional convolutional neural network (2D CNN), with the primary difference being that the convolution operation is performed on one-dimensional sequences. It generally consists of the following components:

The input layer receives one-dimensional sequential data as the model input. The convolutional layer serves as the core of the 1D CNN, which employs one-dimensional convolution kernels to perform convolution operations on the input sequence for extracting local features. The size and number of convolution kernels are critical parameters that determine the granularity of feature extraction and the complexity of the model.

The activation function applies a nonlinear transformation to the output of the convolutional layer, enhancing the representation capability of the network. The pooling layer, usually placed after the convolutional layer, is used to downsample the feature map to reduce data dimensionality, redundant information and computational cost, while improving the robustness and generalization ability of the model. Common pooling operations include max pooling and average pooling.

The fully connected layer, located at the end of the network, conducts global analysis and decision-making on the high-level features extracted by the convolutional and pooling layers. It maps local features to global categories or labels, thereby accomplishing tasks such as classification and regression. The structure of the 1D CNN is illustrated in Figure 3.

### 3.2. GRU Module

In this study, the gated recurrent unit (GRU) module was employed as the main network to process multi-dimensional time-series data, as illustrated in Figure 4. As a typical recurrent neural network (RNN), GRU significantly enhances time-series processing capability by introducing two representative gating mechanisms: the update gate (*z_t_*) and the reset gate (*r_t_*). The update gate determines the extent to which the information stored in the previous time step is retained in the current state. The reset gate controls the proportion of historical information to be forgotten. When the reset gate outputs a value of 0, the model ignores the previous state information and focuses solely on the current input data, which helps the model rapidly adapt to new patterns in the sequence.

### 3.3. Classification Algorithm

A one-dimensional convolutional neural network (1D-CNN) model was adopted for automatic feature extraction and pattern recognition to achieve the classification of the three types of target gases. The response values of the 8 gas sensors were used as inputs, and the target gas species were set as output labels, establishing a mapping relationship between sensor responses and gas components. The sensor features were treated as a one-dimensional numerical sequence, and local correlation features between different sensors were captured via the sliding window scanning of convolution kernels across the sequence.

Local Feature Recognition: The algorithm first reshapes the normalized 8-dimensional sensor data into a one-dimensional sequential input. Two consecutive one-dimensional convolutional layers are configured to perform sliding scanning in the feature space using convolution kernels of varied dimensions. The first layer captures primary local correlations among sensors, while the second layer further extracts higher-order combined features. Through weight sharing, the convolution kernels can sensitively identify the synergistic fluctuation patterns of different sensors under specific gas atmospheres, mapping the original response values into high-dimensional feature maps.

Feature Stabilization and Regularization: To ensure model stability during training, batch normalization is introduced after the convolutional layers. By resetting the neuron outputs to a standard normal distribution, this operation effectively mitigates the vanishing gradient problem commonly encountered in deep networks and significantly accelerates the convergence speed. Meanwhile, the Dropout layer is employed to randomly deactivate a fraction of neurons. By reducing interdependencies between neurons, the model’s generalization ability is enhanced, and overfitting is effectively suppressed.

Global Feature Compression and Fusion: After multi-layer convolutional feature extraction, global average pooling is applied to compress the feature maps globally, which drastically reduces the number of model parameters. This operation aggregates the information of each feature channel into a single scalar representation, retaining global feature information while significantly cutting down the parameter scale. Subsequently, the resulting feature vector is fed into a fully connected layer for nonlinear mapping, achieving comprehensive feature representation and category discrimination.

Probabilistic Decision-Making and Class Balance: At the final output stage of the model, the Softmax activation function is utilized to map the output signals from the preceding layers into a probability distribution over all gas categories, satisfying the mathematical property that the sum of probabilities across all categories equals 1. To address the potential class imbalance in the experimental dataset, a class weight compensation strategy is incorporated during the training phase. By automatically calculating the sample distribution ratio and assigning higher loss weights to minority-class samples, the model ensures equivalent sensitivity in recognizing rare samples, thereby achieving high-precision and balanced identification across all categories.

### 3.4. Prediction Algorithm

A regression prediction architecture based on deep temporal feature mining and multi-task learning is proposed in this study to address the challenges, including sensor cross-sensitivity, signal dynamic drift and transient response noise in the detection of ternary mixed gases. By integrating steady-state recognition logic, a bidirectional gated recurrent unit (Bi-GRU) and a self-attention mechanism, the proposed algorithm realizes high-precision quantitative analysis of mixed gas components.

Data Preprocessing and Feature Engineering: First, a transient response suppression mechanism is established. By monitoring variations in target gas concentration, non-steady-state data generated during concentration switching is automatically identified and filtered, ensuring that the input data are steady-state signals acquired after dynamic equilibrium between gas sensors and measured gases. In terms of feature extraction, differential features are introduced to characterize the dynamic variation trends of signals in addition to original sensor responses. Meanwhile, quantile transformation is applied to standardize nonlinear sensor signals and make them follow a normal distribution, which eliminates the adverse effects of different sensor ranges on model convergence.

Temporal Sliding Window: To fully exploit the historical dynamic information of sensor responses, a temporal sliding window strategy is adopted to convert continuous sensor data into input sequences with temporal dependencies. This approach effectively captures the temporal variation characteristics of sensor responses and improves the model robustness against noise interference.

Deep Neural Network: The core network of this work is constructed based on Bi-GRU. Compared with conventional recurrent neural networks, Bi-GRU extracts features along both forward and reverse time directions, so as to comprehensively capture the evolution rules of sensor arrays in complex gas environments. After temporal feature extraction, a spatial self-attention layer is embedded into the model. This mechanism calculates the contribution of each feature channel and dynamically assigns higher weights to sensors that are most sensitive to target components. Such dynamic weight allocation remarkably enhances the model’s capability of feature separation for mixed gases.

Multi-task Learning and Integrated Training Strategy: A multi-task learning framework is adopted to simultaneously predict the concentrations of three components, namely MEA, MDEA and AMP. The three prediction branches share the underlying feature extractor while maintaining independent output layers. This design enables feature complementation and error compensation among different gas components at the bottom layer. The Huber loss function, which is highly robust to outliers, is employed to balance prediction accuracy and stability. The formula for the Huber loss function is presented in Equation (2).

(2) $$L_{\delta }(y,\;f(x))=\;\{\begin{array}{c} \frac{1}{2}{\left(y-f(x)\right)}^{2},|y-f(x)|\leq \delta \\ \delta (|y-f(x)|-\frac{1}{2}\delta ),|y-f(x)|>\delta \end{array}$$

where *δ* denotes the hyperparameter. The overall block diagram of the classification and prediction algorithms is shown in Figure 5.

### 3.5. Dataset and Preprocessing

#### 3.5.1. Dataset Composition

The experimental dataset consists of response signals acquired by eight sensors for five concentration levels of every single gas, as well as responses of three gases mixed in different proportions. The original classification dataset contained 7783 MEA samples, 9630 MDEA samples, 5472 AMP samples, and 22,531 ternary-mixture samples. Five-fold cross-validation was adopted for model training. Afterward, the entire dataset was split into a training set and a validation set at a ratio of 8:2 and the number of samples in each specific set can be seen in Table S2.

#### 3.5.2. Data Preprocessing

To eliminate the interference of stray data, baseline calibration and normalization were performed on all raw data before feeding into the model. The long-term stability and drift of the commercial sensors were not systematically evaluated in this study. This is a limitation of the present work. However, the differential features used in the pipeline are designed to suppress slow baseline drift, because differencing removes low-frequency components.

Baseline Calibration: Due to long-term heating and material aging, sensors cannot easily return to their initial baseline during operation. To mitigate the influence of baseline drift, all data were first calibrated using Equation (3):

(3) $$S_{baseline}=S_{r}-S_{f}$$

where *S*_r_ represents the raw data value, and *S*_f_ denotes the fitted baseline value.

Normalization: During the measurement, different types of sensors exhibit significant variations in response intensity toward the same gas. Directly inputting the raw response intensities of various sensors into the model may result in deviations in the final prediction due to extremely large or small signal values from individual sensors. Normalization can map the original data to the range of [0, 1] to eliminate such interference. The normalization method is given by Equation (4):

(4) $$S_{i}^{\prime }=\frac{S_{i}-S_{\min}}{S_{\max}-S_{\min}}$$

where *S*_i_ is the raw data, *S*_min_ denotes the minimum value of the dataset, and *S*_max_ denotes the maximum value of the dataset.

#### 3.5.3. Evaluation Metrics

For the classification task, Accuracy (Acc) was adopted as the evaluation metric, which refers to the proportion of correctly predicted samples.

(5) $$Acc=\frac{TP+TN}{TP+TN+FP+FN}$$

where *TP* stands for True Positive, referring to the number of positive samples correctly classified by the classifier. *TN* stands for True Negative, representing the number of negative samples that are correctly classified. *FP* denotes False Positive, meaning the number of negative samples misclassified as positive. *FN* denotes False Negative, referring to the number of positive samples misclassified as negative. For the regression task, the coefficient of determination (R^2^) and root mean square error (RMSE) were used to evaluate the fitting performance between model outputs and experimental data. (R^2^) reflects the goodness of fit between true values and predicted values, which generally ranges from 0 to 1. A value closer to 1 indicates a better fitting effect. RMSE quantifies the prediction accuracy by calculating the average deviation between predicted values and actual values. The corresponding formulas are presented as follows:

(6) $$R^{2}=1-\frac{\sum_{i=1}^{n}{\left(f_{i}-{\hat{f}}_{i}\right)}^{2}}{\sum_{i=1}^{n}{\left(f_{i}-\bar{f}\right)}^{2}}$$

(7) $$RMSE=\sqrt{\frac{1}{n}\sum_{i=1}^{n}{\left(f_{i}-{\hat{f}}_{i}\right)}^{2}}$$

Mean absolute error (MAE) was also adopted. A MAE value closer to 0 indicates better model performance. The corresponding formula is shown below:

(8) $$MAE=\frac{1}{n}\sum_{i=1}^{n}\left|f_{i}-{\hat{f}}_{i}\right|$$

where n is the total number of samples, *f*_i_ denotes the true value, and f represents the predicted value.

## 4. Results and Discussion

### 4.1. Processing of Gas Test Experimental Data

The 1D-CNN was adopted as the classification model in this work. The training and validation curves as well as the classification confusion matrix are shown in Figure 6. As illustrated in Figure 6a,b, the accuracy became stable after 60 training epochs, and the total loss converged to its minimum steady range. No obvious overfitting or gradient explosion was observed, which indicates that the network structure and parameter settings are reasonable.

It can be seen from the confusion matrix in Figure 6c that all samples of the three single-component gases and their mixtures were classified correctly, achieving a classification accuracy of 0.9969, but the main confusion occurred between high-concentration MEA and MDEA, which is chemically reasonable because both are secondary amines with similar dynamic response patterns when one component dominates. The clusters are less separated under non-equal-proportion conditions, especially when the concentration of one amine is much higher than the others. The experimental results verify that the two convolutional layers of the 1D-CNN can effectively extract primary local correlations and high-order combined features among sensor signals. The weight sharing mechanism of convolution kernels accurately captures the synergistic response patterns of different sensors under target gases. This automatic feature extraction method reduces the complexity of manual feature engineering used in traditional machine learning, eliminates errors caused by individual operational differences, and improves classification accuracy while simplifying the overall workflow. The detailed independence-verification results and classification performance are summarized in Table S3.

The concentration of each individual component in mixed gas is critical for in situ monitoring of gas variation. After successfully distinguishing single-component gases and mixtures, concentration prediction was performed for each gas, and the results are presented in Figure 7a–c.

It can be observed that the predicted values are linearly correlated with the true values. The coefficients of determination (R^2^) of MEA, MDEA and AMP are 0.9988, 0.9946 and 0.9973, respectively, as summarized in Table 3. Figure 7d–f shows the dynamic tracking curves of predicted concentrations. The predicted values maintain high consistency with the actual values for all three gases, which further demonstrates the excellent effectiveness and robustness of the proposed model for concentration prediction. The key hyperparameter settings are summarized in Table S4, which lists the main configurations of both the 1D-CNN classification model and the proposed concentration prediction model, including the network architecture, kernel size, hidden units/filters, learning rate, window length, optimizer, batch size, loss function, and maximum number of training epochs.

Table 3 compares the evaluation metrics for concentration prediction of the three gases. MEA achieves the optimal performance with the highest R^2^ and the lowest RMSE and MAE values, followed by AMP. MDEA delivers slightly poorer prediction results for individual component concentrations in mixed gas, which is attributed to the similar molecular structures of MEA and MDEA. Overall, the prediction performance for all three gases is satisfactory.

The MOS sensors employed in this study are broadly cross-sensitive rather than chemically specific. Therefore, the identification and concentration prediction of MEA, MDEA, and AMP rely on the combined response pattern of the sensor array instead of the response of a single sensor, so we conducted an additional sensor-level interpretability analysis using Spearman correlation and channel-wise permutation importance. Spearman correlation was employed to quantify the monotonic relationship between each sensor response (S1–S8) and the concentration of each analyte. Channel-wise permutation importance was used to evaluate the contribution of each sensor channel to the trained prediction model. Specifically, one sensor channel was randomly permuted across the test samples while the remaining seven channels were kept unchanged. Because both the original sensor responses and their first-order differential features were included in the model input, each original sensor channel and its corresponding differential channel were permuted simultaneously using the same sample order. The permutation procedure was repeated 20 times for each sensor channel in each of the five folds; the results are shown in Figure S2.

### 4.2. Ablation Experiment

To investigate the contribution and importance of each module to the organic amine prediction task, we performed an ablation study on different modules and their combinations. The experimental results can be seen in Table 4, which were consistent with our expectation that more modules bring better prediction performance. The self-attention module can provide lower values of RMSE, MAE and MAPE. With the change in the multi-task learning framework, the performance of the sensor array will undergo tremendous changes. The value of R^2^ continues to increase, owing to the sharing of parameters among different tasks. The ablation study results for different modules and visualized attention weights can iteratively bring crucial guiding significance for organic amines recognition and concentration prediction strategies in application-specific sensing tasks.

### 4.3. Comparison of Different Classification Methods

The results of the feature-based methods and the deep learning-based method with five-fold input data are shown in Table 5. Handcrafted feature extraction for Gaussian NB, AdaBoost, LR and SVM was used for classification, and four representative mainstream deep-learning architectures were also used, including LSTM, CNN-LSTM and TC. To ensure a consistent comparison, all additional deep-learning baselines were evaluated using the same classification dataset, preprocessing procedure, and five-fold cross-validation protocol as the proposed model. Standard baseline configurations with commonly used hyperparameter settings were adopted to ensure a fair comparison while avoiding excessive model-specific tuning. The results indicate that five-fold cross-validation is not beneficial for feature-based methods. For instance, the accuracy of Gaussian NB and AdaBoost is only 0.7462 ± 0.005 and 0.7594 ± 0.089, respectively. Furthermore, the proposed deep-learning-based method, which combines a CNN with 1D input data, achieves an average precision of 0.9969 ± 0.0006 and outperforms the best feature-based method, SVM (which has an average precision of 0.9950 ± 0.001). The LSTM model achieved an Accuracy of 0.9585 ± 0.0062 and an F1-score of 0.9588 ± 0.0060. CNN-LSTM improved the Accuracy to 0.9806 ± 0.0077, while TCN further achieved 0.9873 ± 0.0067. Nevertheless, the proposed model still achieved the highest values for all four classification metrics: Accuracy, Precision, Recall, and F1-score. Moreover, the zero standard deviation across the five folds indicates highly consistent classification performance under the present experimental conditions. These results demonstrate that the proposed method not only outperforms conventional machine-learning approaches, but also demonstrates improved performance compared with representative deep-learning architectures under the current experimental conditions. However, the concentration intervals in the current dataset are relatively large. Therefore, the high classification accuracy may partly reflect the simplicity of the dataset. Classification performance at ambiguous concentration boundaries remains to be investigated.

### 4.4. Device-Level Sensing Mechanism and Its Role in Recognition

The sensors used in this study are commercial off-the-shelf devices. The manufacturer does not disclose the detailed material composition, microstructure, or morphology. According to the datasheet, the sensors are metal-oxide semiconductor type. Therefore, the mechanistic discussion is limited to the device level and to generally accepted mechanisms for this class of sensors: Target gas molecules interact with pre-adsorbed oxygen species on the sensing surface, leading to charge transfer and a change in the depletion layer, which is transduced into a resistance change. Under temperature modulation, the kinetics of adsorption, surface reaction, and desorption differ among analytes due to differences in molecular structure, bond energy, and reaction activation energy. These kinetic differences produce distinct dynamic response waveforms [21,22,23]. The pattern recognition algorithm (1D-CNN combined with Bi-GRU) exploits exactly these chemically rooted dynamic features: Bi-GRU captures the variance structure associated with these kinetic differences, and 1D-CNN classifies based on the resulting feature space.

### 4.5. Hardware Device and In-Site Industrial Cases Testing Results

In this study, a compact and smart organic amine monitoring device with a gas sensor array and a temperature/humidity detection module was designed and implemented. The STM32F103C8T6 microcontroller is the primary control unit, which features a 32-bit ARM Cortex-M3 core, 64 KB flash and a 10 × 12b A/D developed by STMicroelectronics. Data transmission between the monitoring device and the laptop computer was accomplished through a 433 MHz LoRa RF module pair, which facilitated wireless duplex communication. A 6-inch touch screen was used to display the status of the sensors and the temperature and humidity values. Additionally, the device was designed with a DC circuit and an onboard lithium battery power management circuit, enabling the system to work for a long time and remain in a working state without external power sources for 1–2 h. Figure 8a–c illustrate the digital photo of the PCB of the sensor array, 3D model of the device and the overall physical appearance of the device, respectively. As shown, the device’s layout is compact and fully functional, measuring just 300 mm × 200 mm × 150 mm, which facilitates integration into other systems. Figure 8d shows the predicted concentration trends of the three organic amines in in situ industrial case testing, and the computer interface and the response curves of sensors can be seen in Figures S4 and S5 in the Supplementary Materials. To quantitatively investigate whether all eight sensor channels were necessary, we further conducted a sensor-number ablation experiment using subsets containing 2, 4, 6, and 8 sensors. The sensor subsets were selected according to the channel-importance analysis, and the same data partitioning, preprocessing procedure, model architecture, and evaluation protocol were used in all cases to ensure a fair comparison. The sensors have different sensing materials and cross-sensitivity patterns, and the sensor-number ablation results show that removing sensor channels generally increases the overall prediction error, which can be seen in Figure S6. Therefore, although the sensor channels contain substantial shared information, the complete eight-channel array still provides the best overall prediction performance. The purpose of this experiment was primarily to verify the operational feasibility and on-site response of the integrated hardware system in a practical environment. In future work, simultaneous sampling and comparison with a validated reference method, such as gas chromatography, will be conducted to further evaluate the accuracy, selectivity, and long-term reliability of the device under industrial operating conditions. The hardware circuit design, debugging, and firmware programming of the organic amine monitoring device were all completed by the authors of this paper.

## 5. Conclusions

In this study, a monitoring system for small-molecule organic amines based on a MOS sensor array was successfully developed. By integrating the 1D-CNN and Bi-GRU deep learning models, accurate classification and quantitative concentration prediction of three types of small-molecule organic amine gases and their mixtures were realized. The model was trained using five-fold cross-validation. The 1D-CNN classification model achieved a classification accuracy of 0.9969 ± 0.0006. For the Bi-GRU concentration prediction model, the coefficients of determination (R^2^) for MEA, MDEA and AMP reached 0.9969, 0.9946 and 0.9973, respectively.

In future work, the proposed system will be deployed in practical carbon dioxide capture sites for in situ monitoring of small-molecule organic amines. Further field tests will be conducted to evaluate the comprehensive performance and stability of the system, laying a solid foundation for its large-scale engineering application and popularization.

## Figures and Tables

**Figure 1:**
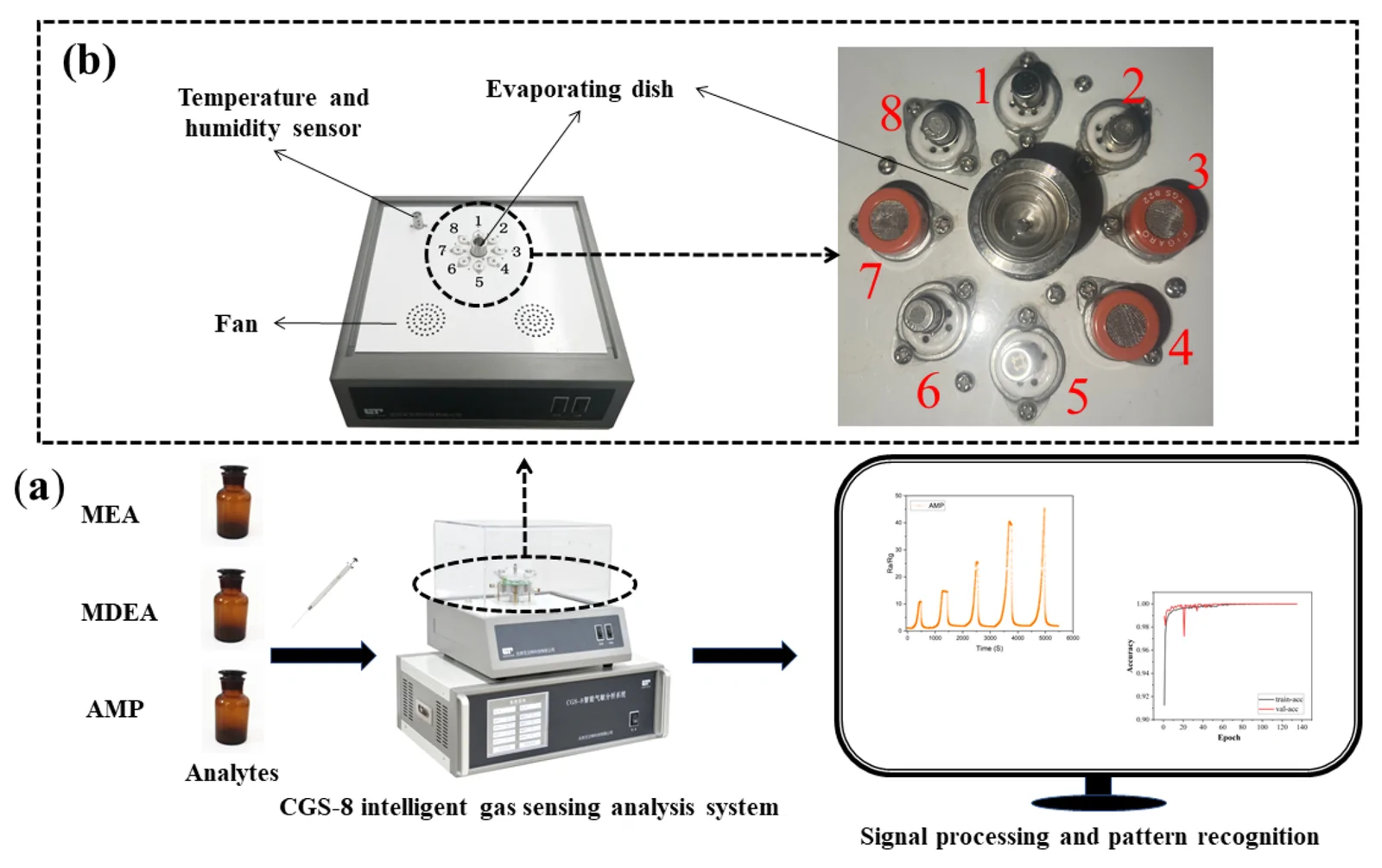
Details of the gas sensing experiment. (**a**) the experimental platform and procedure of gas testing; (**b**) the details of the experimental platform and the number of the sensor sockets.

**Figure 2:**
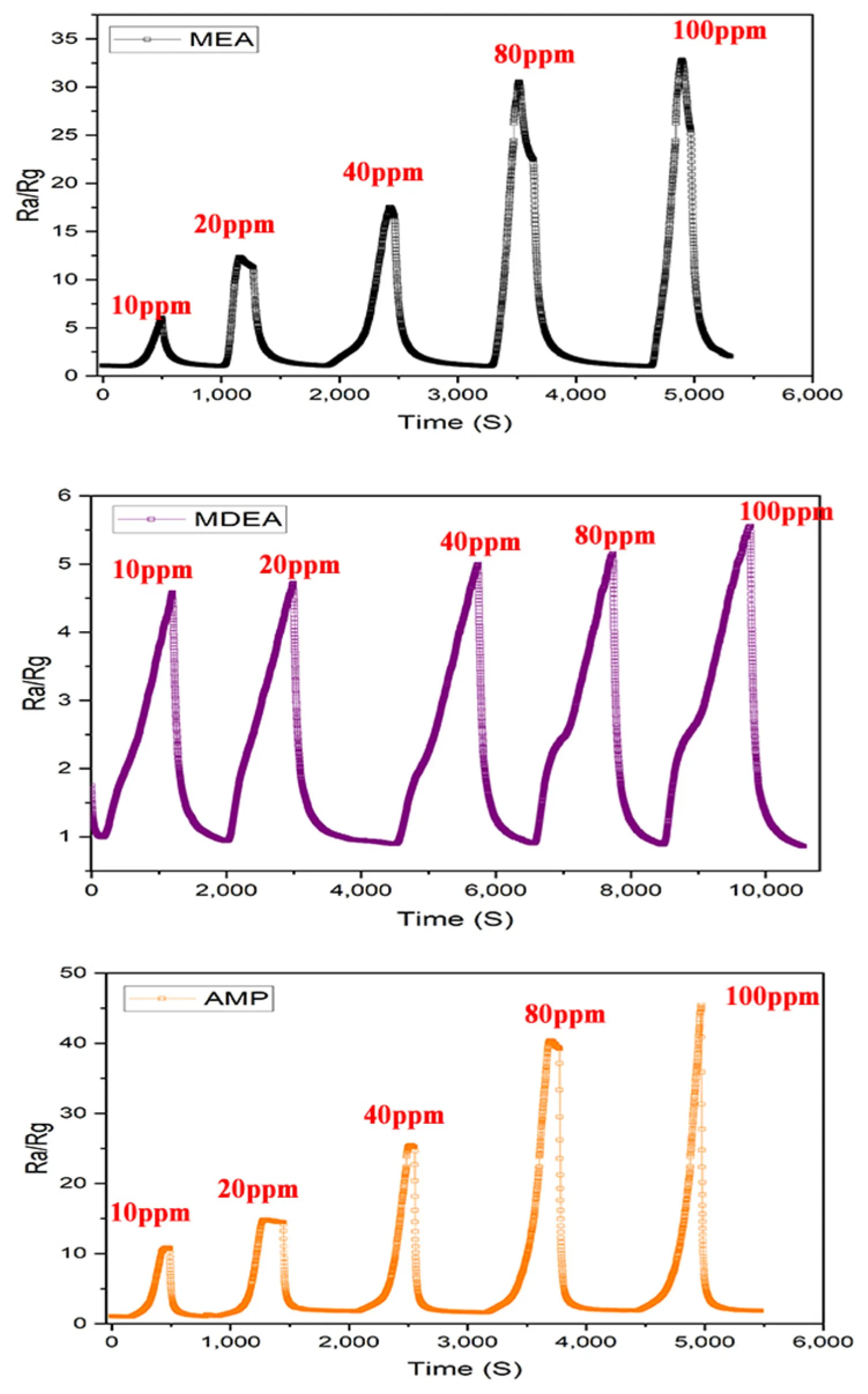
The dynamic curves of three gases with the MP503 sensor.

**Figure 3:**
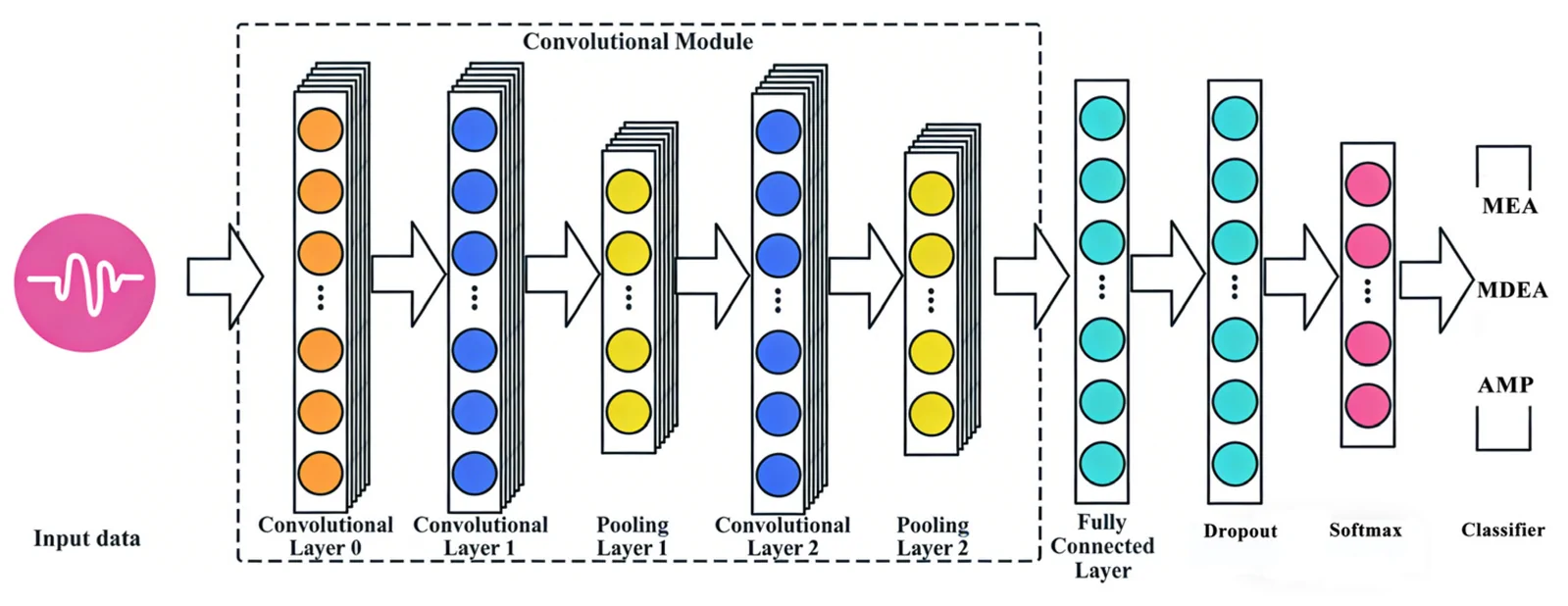
The typical 1D CNN structure for classification of MEA, MDEA and AMP.

**Figure 4:**
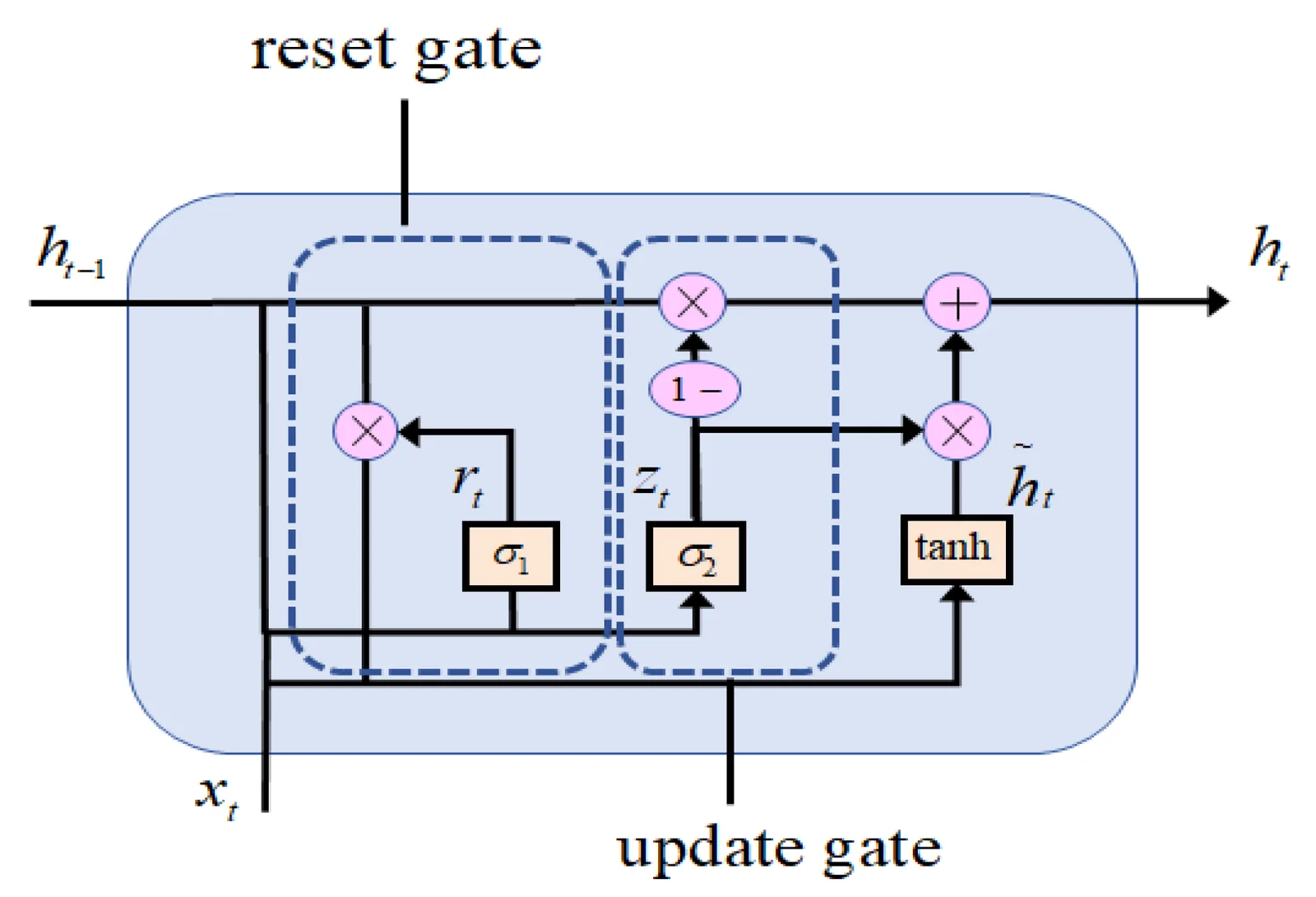
A block diagram of a typical GRU model structure, including a reset gate ($r_{t}$) and an update gate ($z_{t}$).

**Figure 5:**
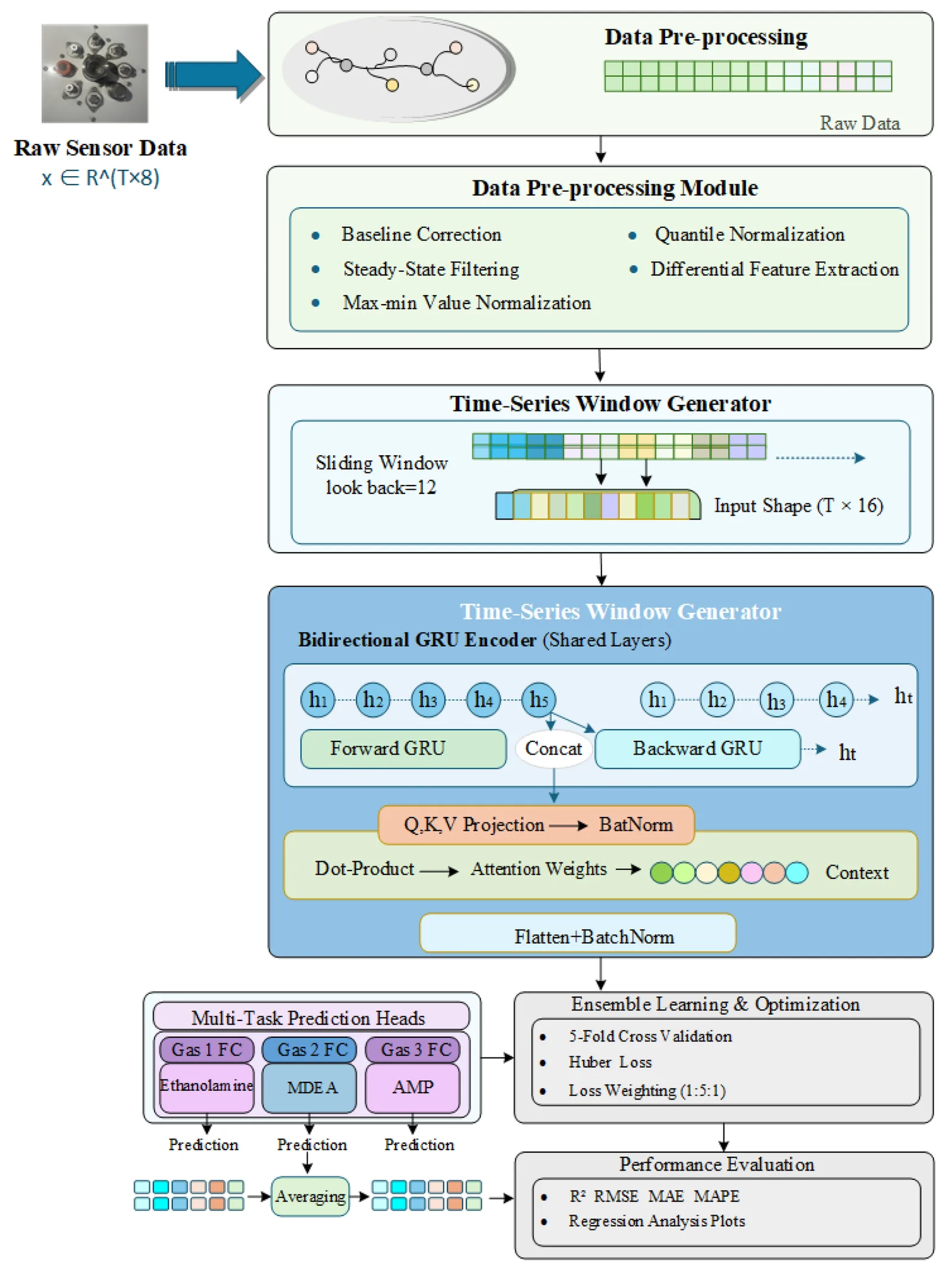
The whole architecture diagram of prediction.

**Figure 6:**
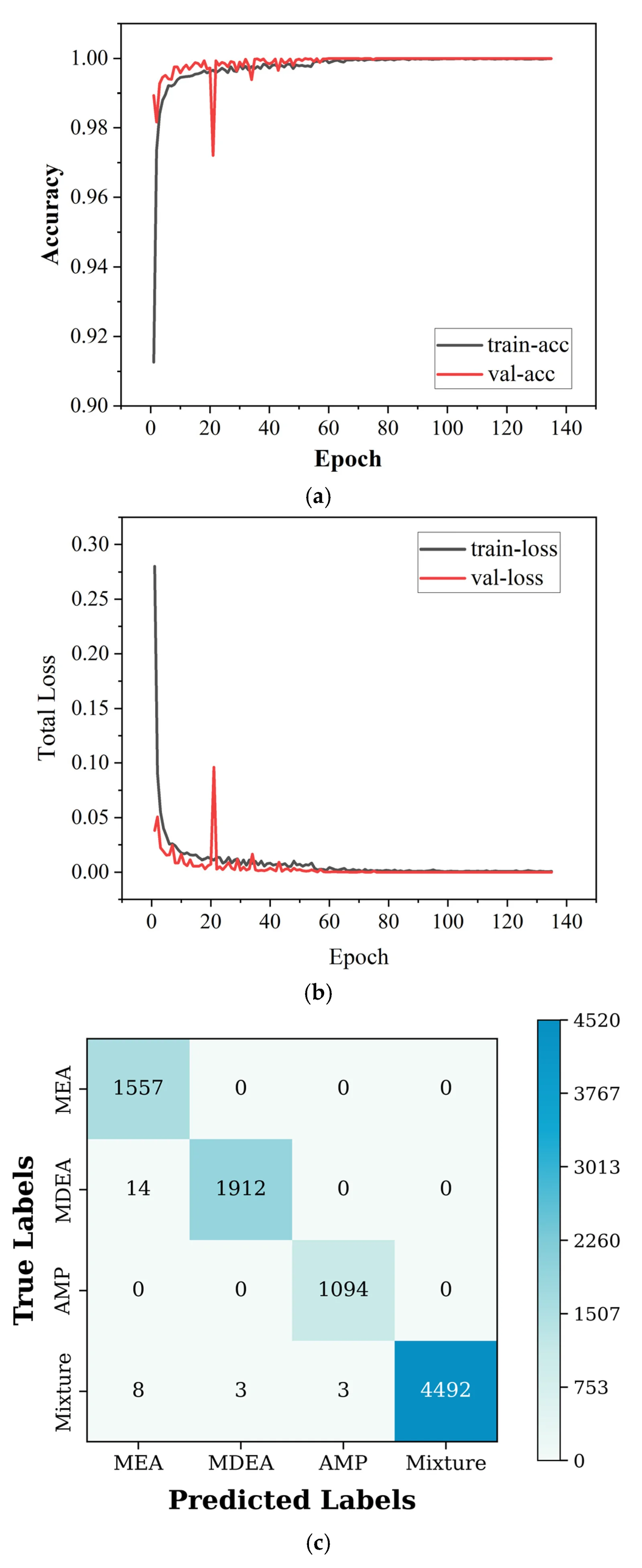
Training and validation performance of 1D-CNN model structures: (**a**) accuracy curve, (**b**) total loss curve, (**c**) confusion matrix.

**Figure 7:**
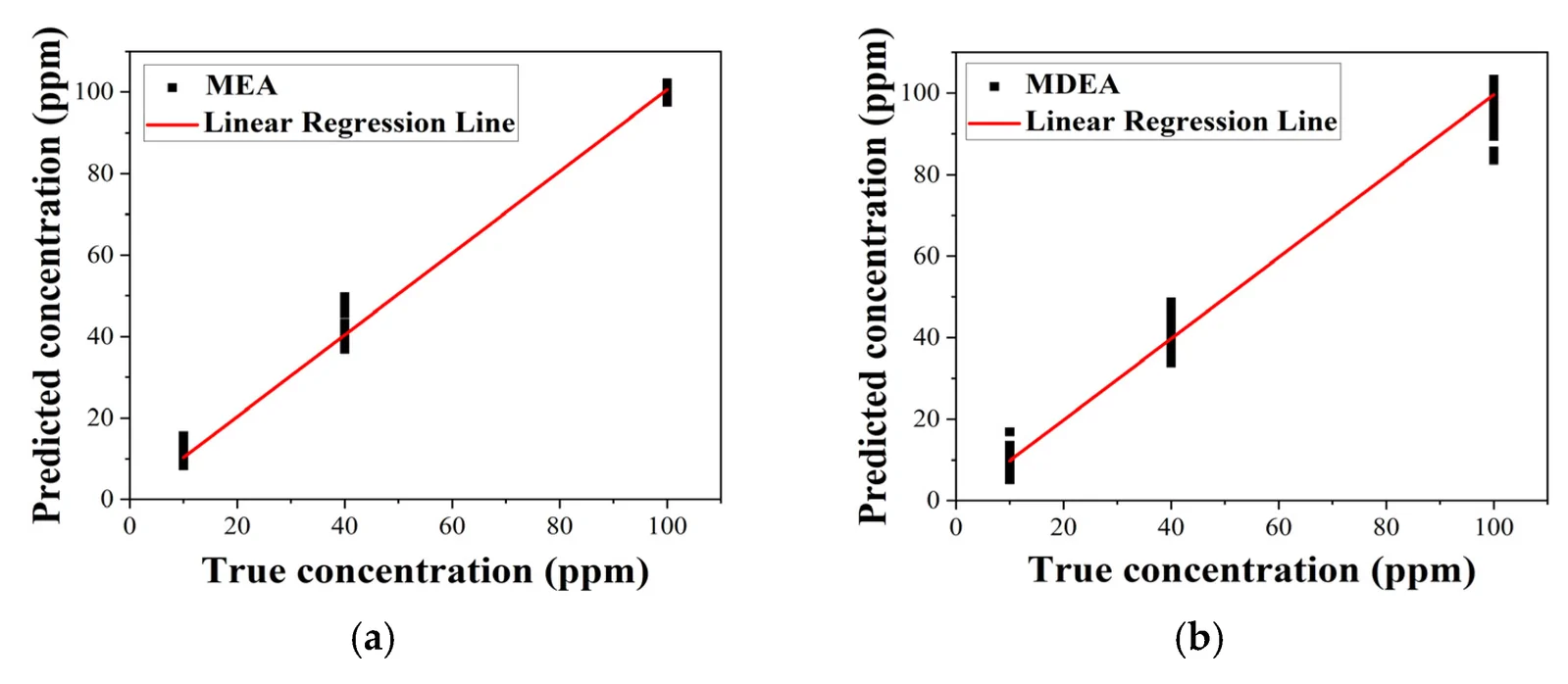

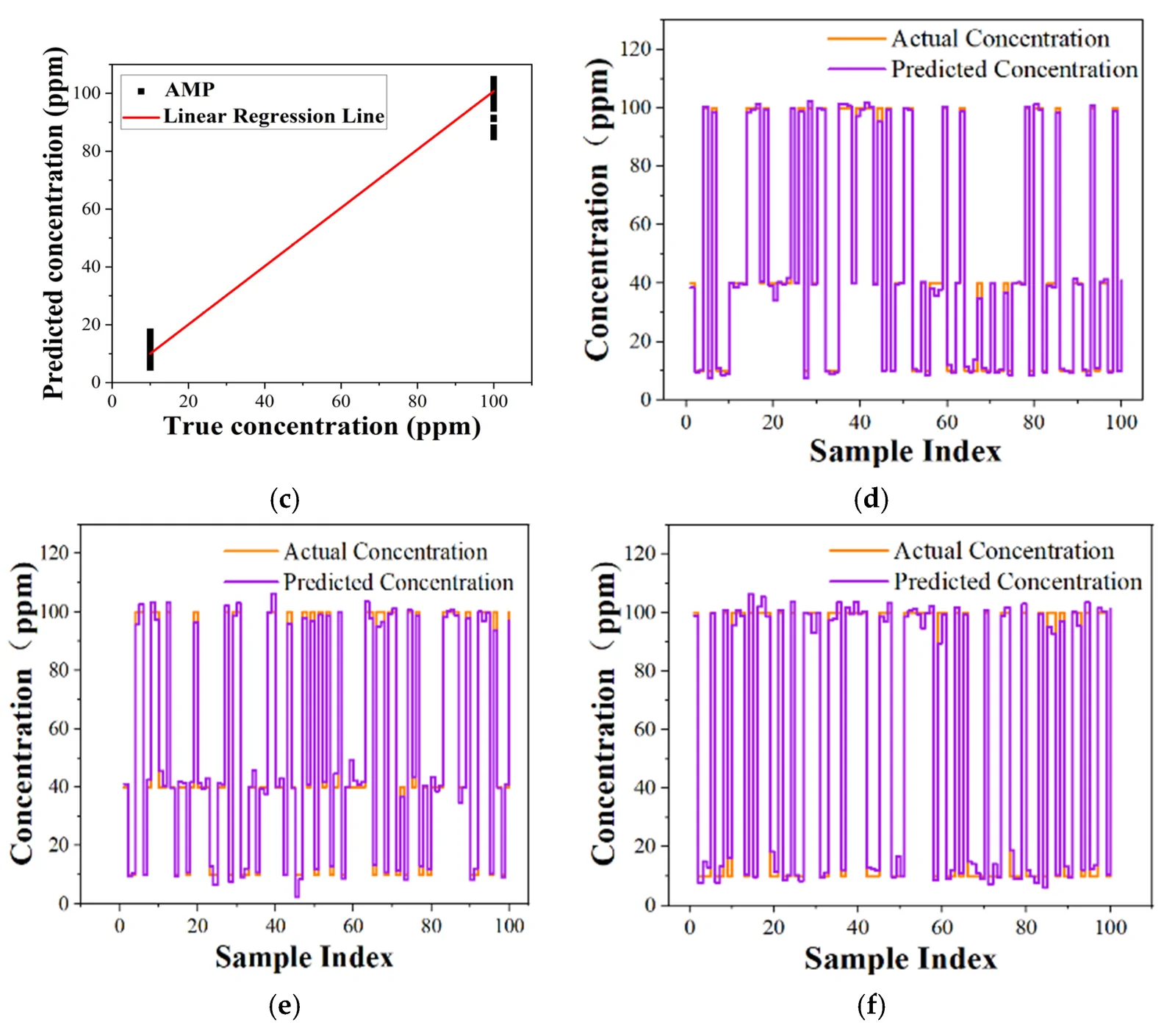
Linear fitting curves of three gases in a mixed gas and dynamic following curves: the linear fitting curves of (**a**) MEA, (**b**) MDEA, (**c**) AMP and the dynamic following curves of (**d**) MEA, (**e**) MDEA, (**f**) AMP.

**Figure 8:**
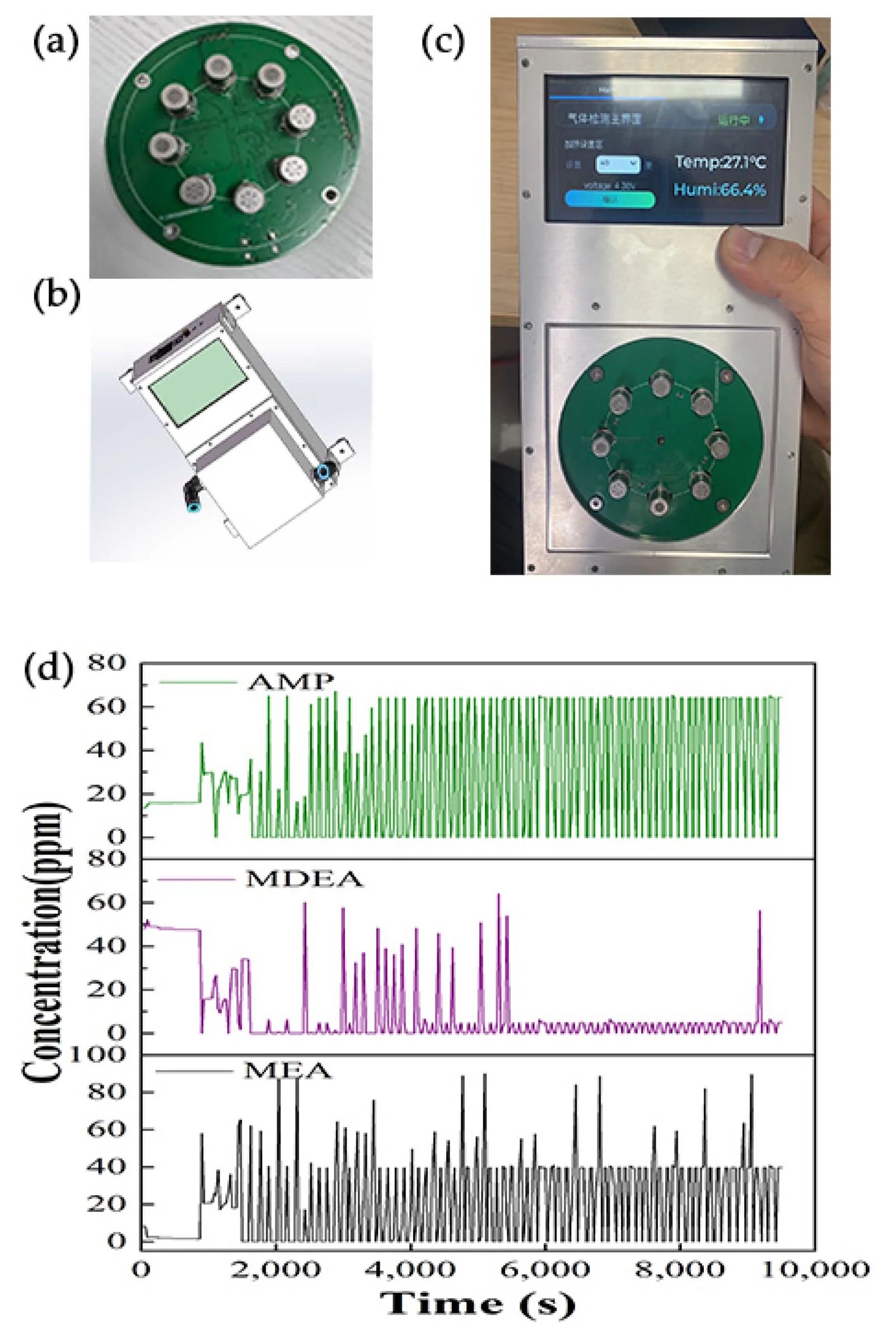
(**a**) the digital photo of the sensor array, (**b**) the 3D model of the device, (**c**) the overall physical appearance of the device with 300 mm × 200 mm × 150 mm, the Chinese characters means Gas Detection Main Interface Running, (**d**) the predicted concentration trends of the three organic amines in-site industrial case testing.

**Table 1:** Parameters of the selected sensors.

Sensor Model	Target Gases	Detection Range (ppm)	Optimum Operating Current (mA)
TGS822	Ethanol, Organic solvents	Ethanol 50–5000	120
TGS2602	VOC, ammonia, hydrogen sulfide	Ethanol 1–30	50
TGS2600	Ethanol, Hydrogen	Hydrogen 1–30	42
MP503	Formaldehyde, Isobutane, Ethanol	Ethanol 1–1000	40
MQ3	Ethanol	Ethanol 25–500	130
MQ135	Ammonia, Benzene, Sulfide	Ammonia, benzene 10–1000	43

**Table 2:** Concentrations of target gases.

Target Gases	Concentration (ppm)
MEA	10, 20, 40, 80, 100
MDEA	10, 20, 40, 80, 100
AMP	10, 20, 40, 80, 100
Mixture (MEA:MDEA:AMP)	10:10:10, 10:40:10, 10:40:100, 10:100:10, 10:10:100, 10:100:100, 40:10:10, 40:40:10, 40:100:10, 40:10:100, 40:40:100, 40:100:100, 100:10:10, 100:40:10, 100:100:10, 100:10:100, 100:40:100, 100:100:100

**Table 3:** Comparison of evaluation metrics for prediction results of three gases.

Gas Type	R^2^	RMSE	MAE
MEA	0.9969	2.1306	1.5503
MDEA	0.9946	2.7534	2.0775
AMP	0.9973	2.3276	1.6836

**Table 4:** Ablation study results of the prediction model.

Model	R^2^	RMSE	MAE	MAPE (%)
Bi-GRU	0.9073	11.4504	8.1250	41.3842
Bi-GRU + Attention	0.9919	3.4085	2.2333	10.8865
Bi-GRU + Attention + MTL (MSE loss)	0.9945	2.8452	1.9250	8.3338
Proposed Model	0.9969	2.1306	1.5503	7.3043

**Table 5:** Comparison Results of Different Classification Models.

Model	Accuracy	Precision	Recall	F1
Gaussian NB	0.7462 ± 0.005	0.7544 ± 0.006	0.7462 ± 0.005	0.7454 ± 0.006
AdaBoost	0.7594 ± 0.089	0.8013 ± 0.038	0.7594 ± 0.090	0.7637 ± 0.074
Logistic Regression	0.9778 ± 0.002	0.9795 ± 0.002	0.9778 ± 0.002	0.9780 ± 0.002
SVM	0.9949 ± 0.001	0.9950 ± 0.001	0.9949 ± 0.001	0.9949 ± 0.001
LSTM	0.9585 ± 0.0062	0.9595 ± 0.0057	0.9585 ± 0.0062	0.9588 ± 0.0060
CNN-LSTM	0.9806 ± 0.0077	0.9818 ± 0.0069	0.9806 ± 0.0077	0.9807 ± 0.0077
TCN	0.9873 ± 0.0067	0.9878 ± 0.0063	0.9873 ± 0.0067	0.9874 ± 0.0067
Proposed Model	0.9969 ± 0.0006	0.9970 ± 0.0006	0.9969 ± 0.0006	0.9969 ± 0.0006

Note: Classification results are reported as mean ± standard deviation over five folds.

## Data Availability

The original contributions presented in this study are included in the article/Supplementary Materials. Further inquiries can be directed to the corresponding authors.

## Supplementary Materials

The following supporting information can be downloaded at: https://www.mdpi.com/article/10.3390/s26196012/s1, Figure S1. the response curves of MEA, MDEA and AMP from 10ppm to 100ppm of (a) TGS2600-1, (b) TGS2600-2, (c) TGS822-1, (d) TGS822-2, (e) TGS2620, (f) MQ3 and (g) MQ135. Figure S2. Dynamic response curve of the MP503 sensor under 18 ternary-mixture concentration combinations. C1-C18 correspond to the concentration combinations listed in Table S1. Figure S3. Sensor-level interpretability analysis: (a) Spearman correlations between individual sensor responses and analyte concentrations; (b) normalized channel-wise permutation importance for the prediction of MEA, MDEA, and AMP. Figure S4. The on-site testing results of the response curve of sensors array. Figure S5. The software interface of on-site testing. Figure S6. Sensor-number ablation and multicollinearity analysis: (a) RMSE values obtained using 2, 4, 6, and 8 sensor channels; (b) Pearson correlation matrix of the eight sensor channels. Table S1. Concentration combinations of ternary mixtures (MEA:MDEA:AMP, ppm). Table S2. Distribution of samples in the training and independent test sets. Table S3. Independence verification and classification performance on the independent test set. Table S4. Hyperparameter settings of the classification and prediction models.
