# Supplementary material for: Hybrid Deep Learning Neural Networks for Small-Molecule Organic Amine Gas Recognition and Prediction Based on MOS Sensor Array
Source: Sensors (Basel). 2026 Sep 23;26(19):6012. doi: 10.3390/s26196012 (PMC13667567; doi:10.3390/s26196012)
Supplement: Supplementary file 1 [file sensors-26-06012-s001.zip › sensors-4547374-supplementary.pdf]

Supporting Information

(a)

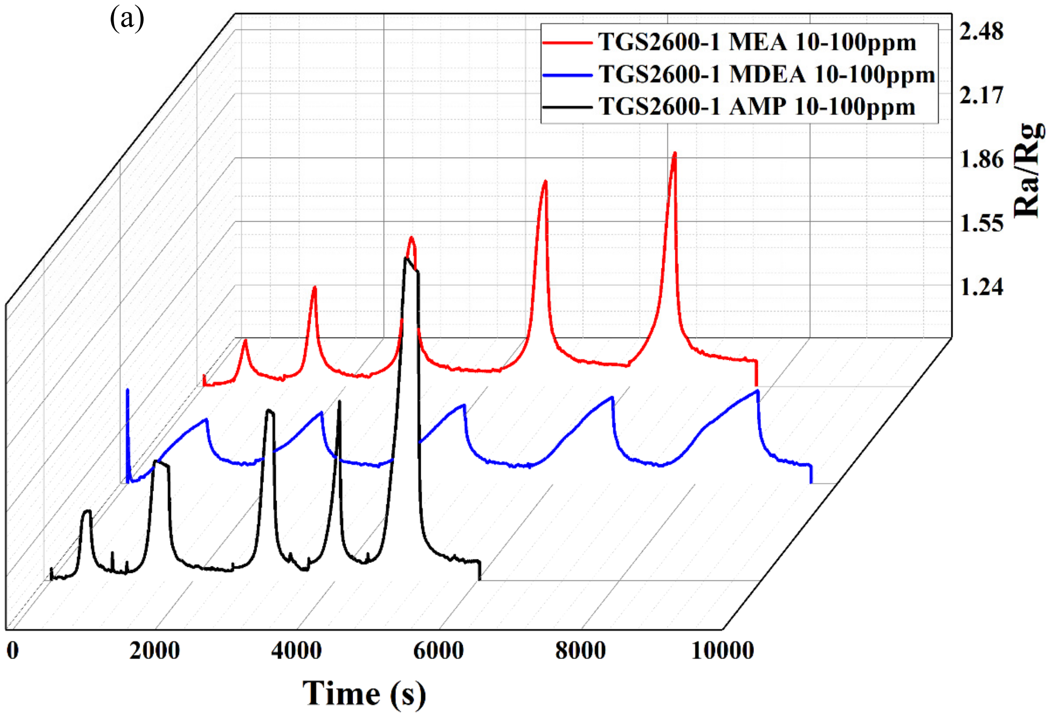

(b)

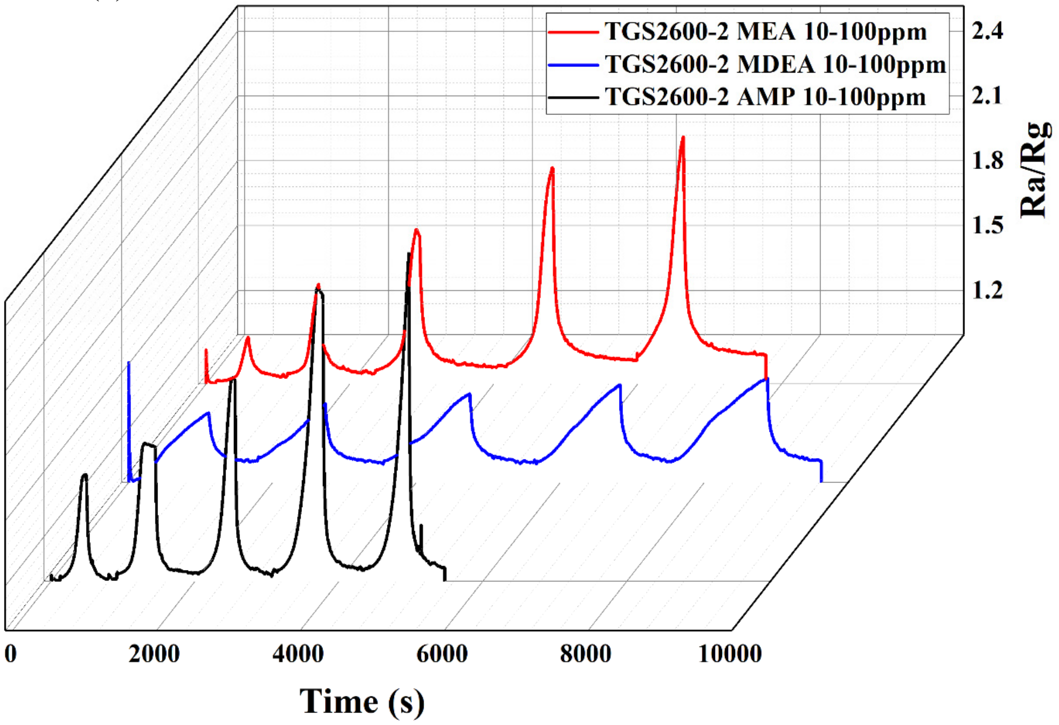

(c)

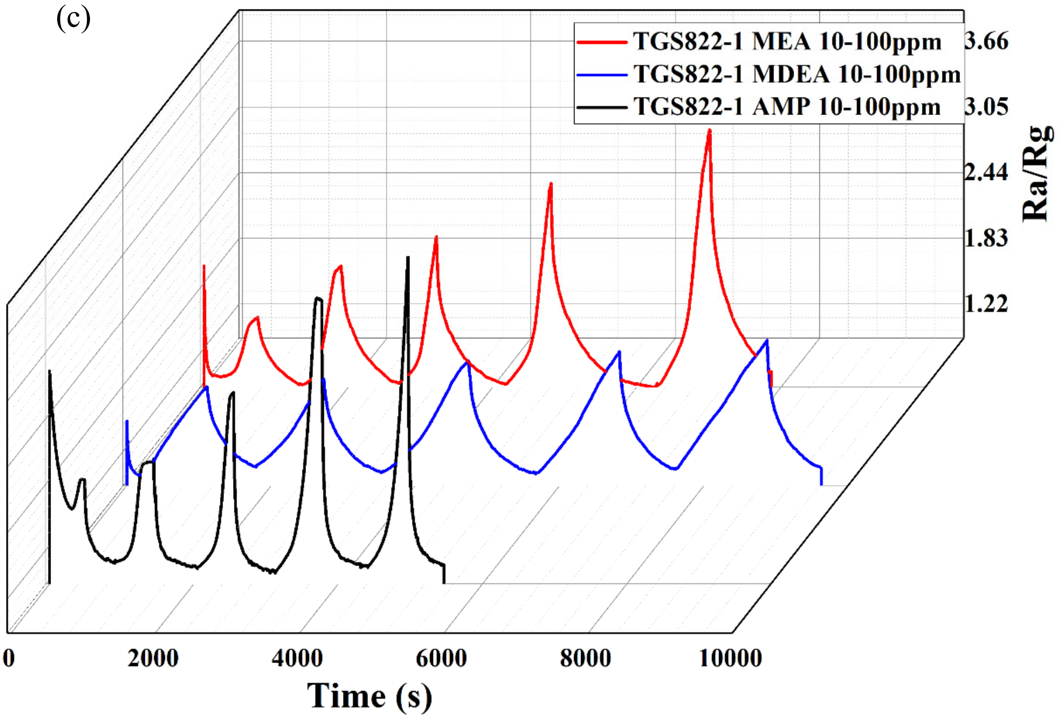
TGS822-1 MEA 10-100ppm
TGS822-1 MDEA 10-100ppm
TGS822-1 AMP 10-100ppm
3.66
3.05
2.44
1.83
1.22
Ra/Rg
0
2000
4000
6000
8000
10000
Time (s)

(d)

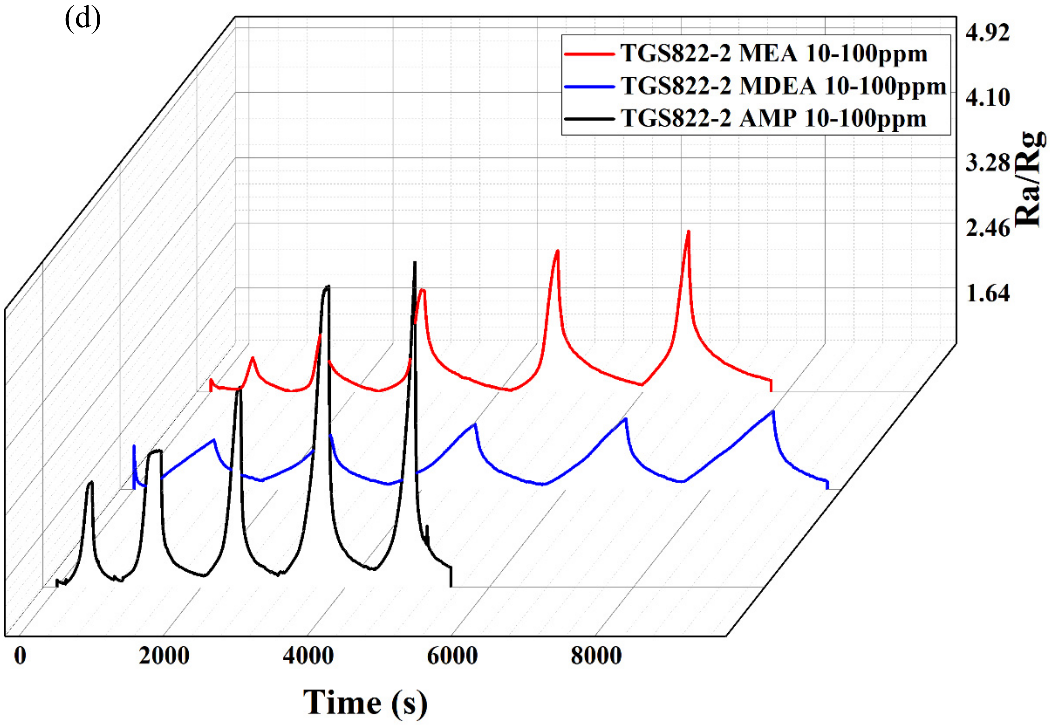
TGS822-2 MEA 10-100ppm
TGS822-2 MDEA 10-100ppm
TGS822-2 AMP 10-100ppm
4.92
4.10
3.28
2.46
1.64
Ra/Rg
0
2000
4000
6000
8000
Time (s)

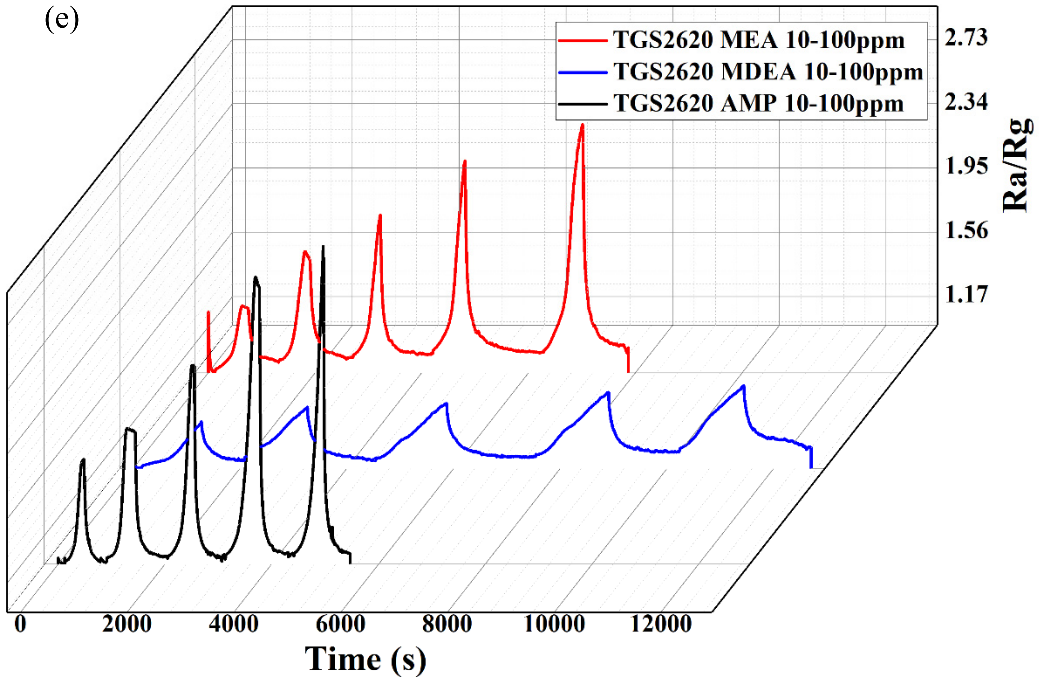

(e)
TGS2620 MEA 10-100ppm
TGS2620 MDEA 10-100ppm
TGS2620 AMP 10-100ppm
2.73
2.34
1.95
1.56
1.17
Ra/Rg
0
2000
4000
6000
8000
10000
12000
Time (s)

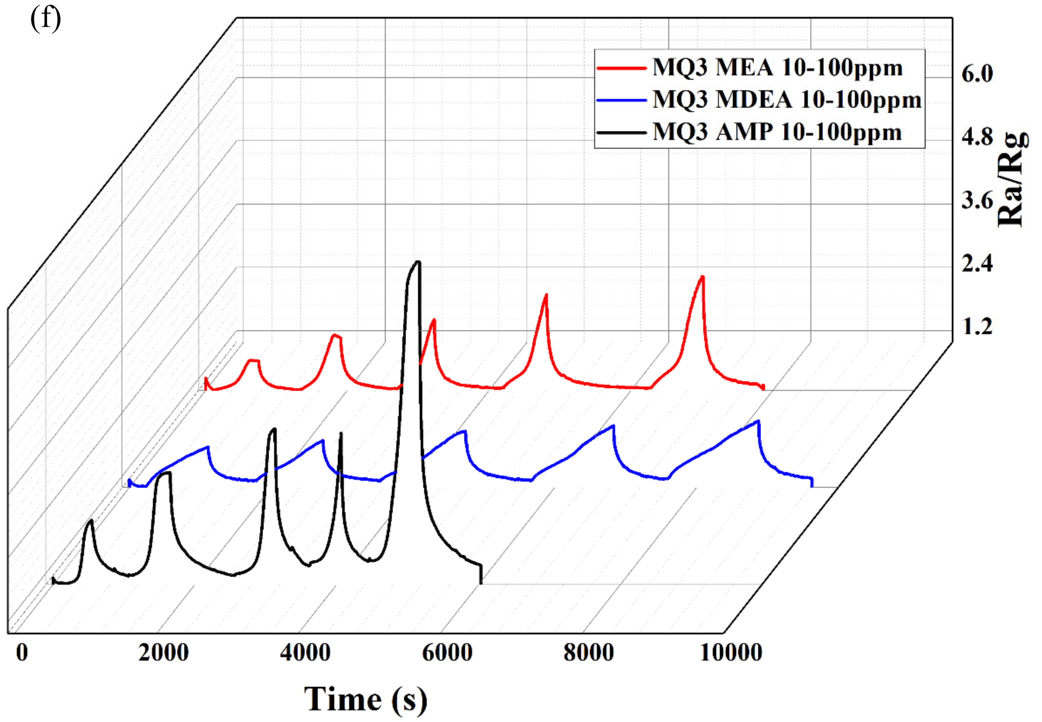

(f)
MQ3 MEA 10-100ppm
MQ3 MDEA 10-100ppm
MQ3 AMP 10-100ppm
6.0
4.8
3.6
2.4
1.2
Ra/Rg
0
2000
4000
6000
8000
10000
Time (s)

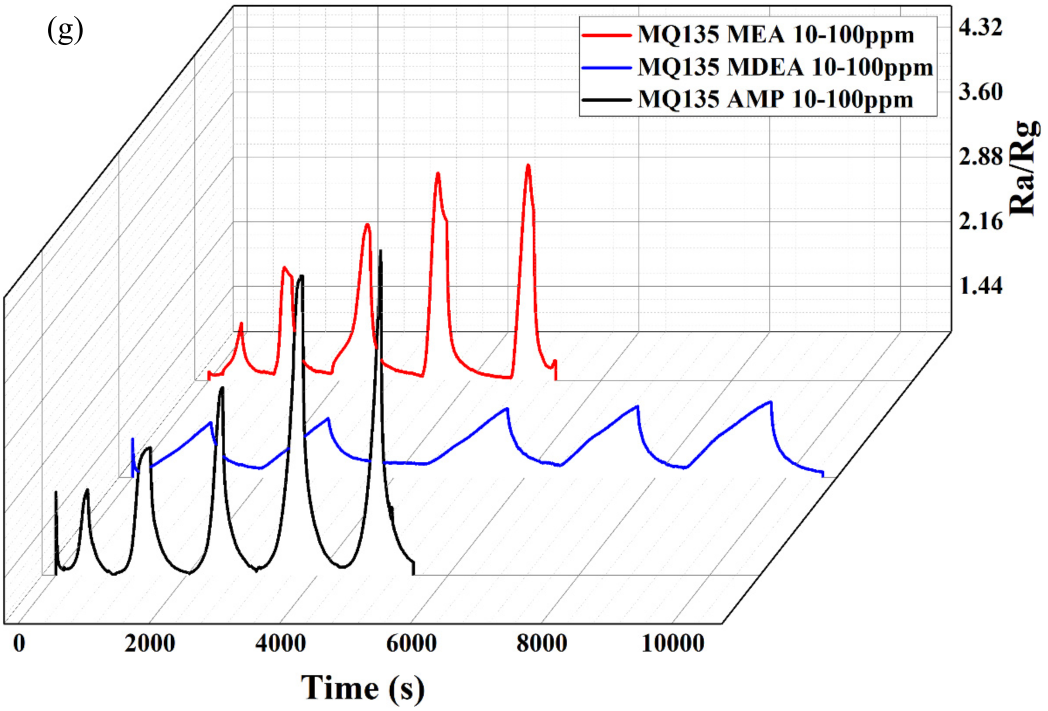

Figure S1. the response curves of MEA, MDEA and AMP from 10ppm to 100ppm of (a) TGS2600-1, (b) TGS2600-2, (c) TGS822-1, (d) TGS822-2, (e) TGS2620, (f) MQ3 and (g) MQ135.

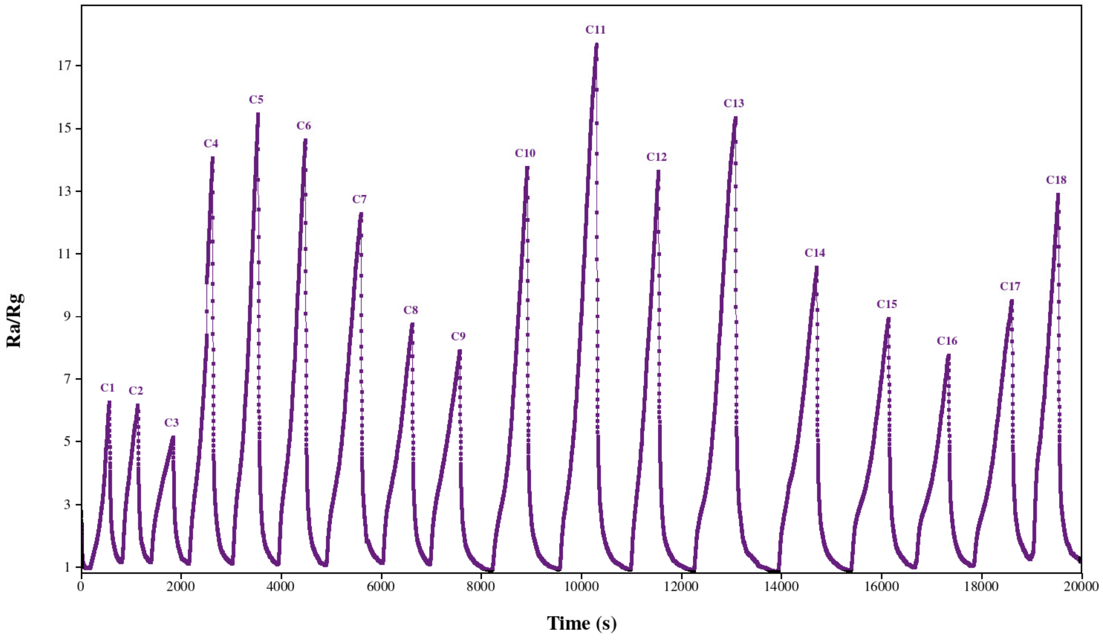

Figure S2. Dynamic response curve of the MP503 sensor under 18 ternary-mixture concentration combinations. C1-C18 correspond to the concentration combinations listed in Table S1.

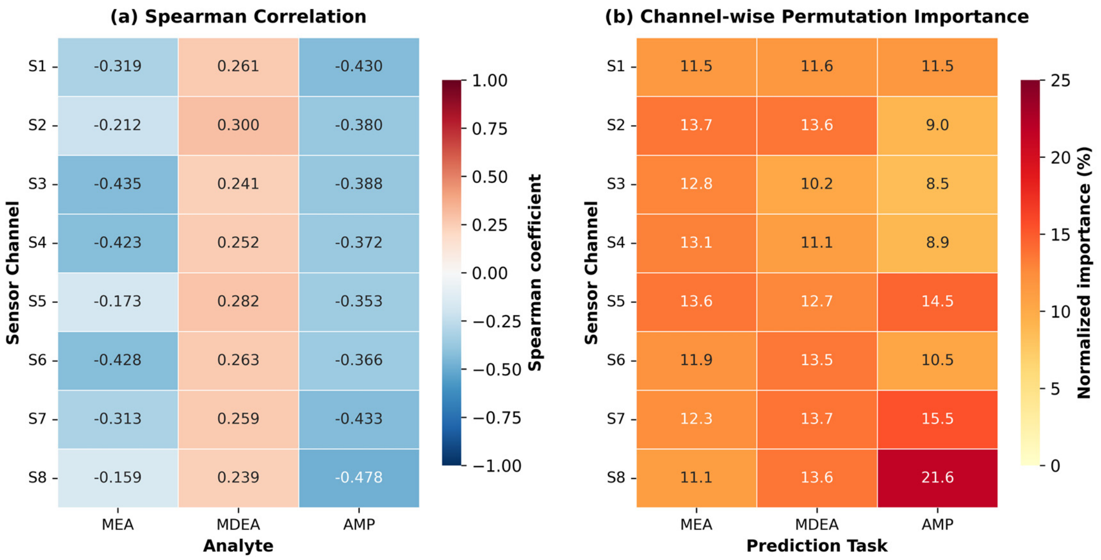

Figure S3. Sensor-level interpretability analysis: (a) Spearman correlations between individual sensor responses and analyte concentrations; (b) normalized channel-wise permutation importance for the prediction of MEA, MDEA, and AMP.

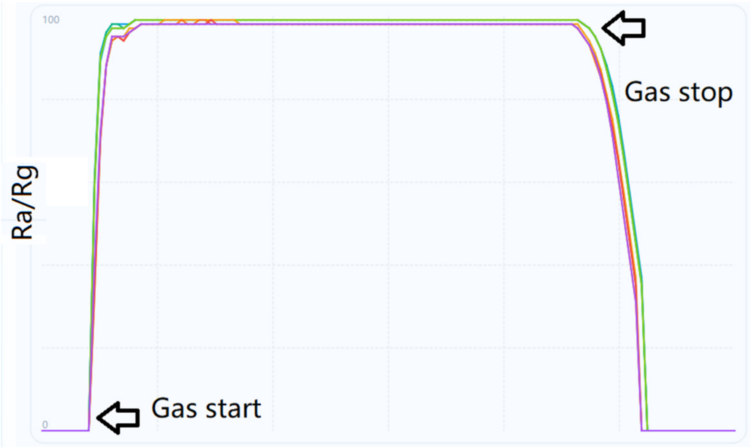

Figure S4. The on-site testing results of the response curve of sensors array.

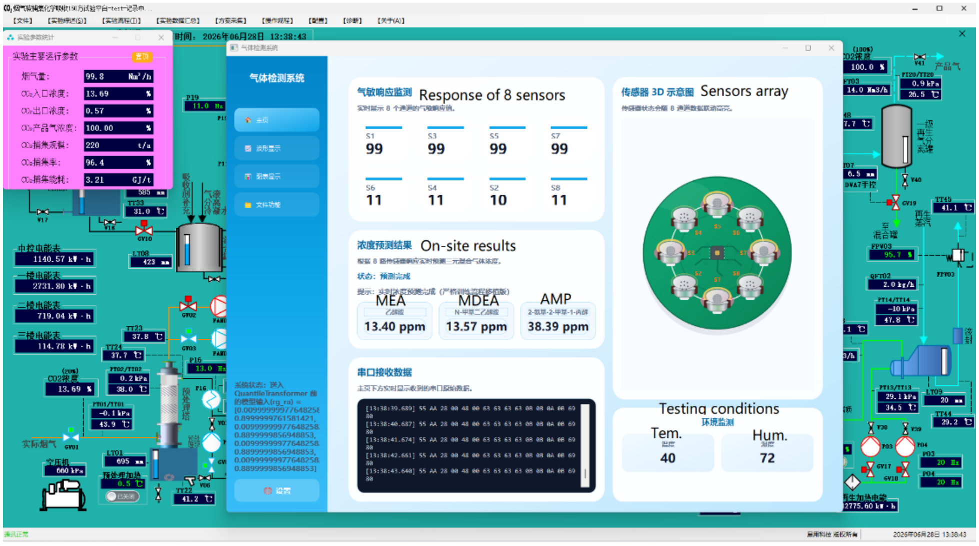

Figure S5. The software interface of on-site testing.

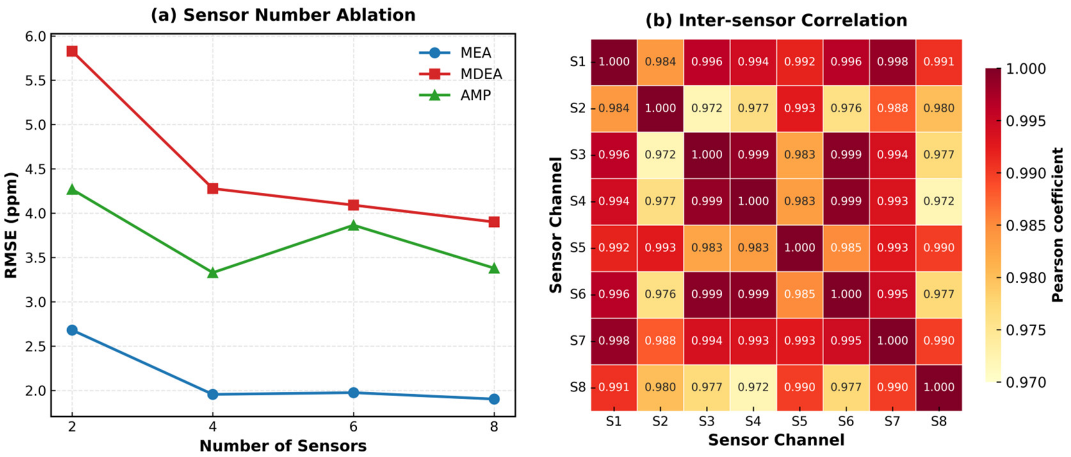

Figure S6. Sensor-number ablation and multicollinearity analysis: (a) RMSE values obtained using 2, 4, 6, and 8 sensor channels; (b) Pearson correlation matrix of the eight sensor channels.

Table S1. Concentration combinations of ternary mixtures (MEA:MDEA:AMP, ppm).

No.	Concentration	No.	Concentration	No.	Concentration
C1	10:10:10	C7	40:10:10	C13	100:10:10
C2	10:10:10	C8	40:10:10	C14	100:10:10
C3	10:100:10	C9	40:10:10	C15	100:10:10
C4	10:100:10	C10	40:10:10	C16	100:10:100
C5	10:40:100	C11	40:10:10	C17	100:40:100
C6	10:40:100	C12	40:10:10	C18	100:40:100

Table S2. Distribution of samples in the training and independent test sets.

Gas category	Total samples	Training set (80%)	Independent test set (20%)
MEA	7,783	6,226	1,557
MDEA	9,630	7,704	1926
AMP	5,472	4,378	1094
Mixture	22,531	18,025	4506
Total	45,416	36,333	9083

Table S3. Independence verification and classification performance on the independent test set.

Item	Result
Total number of samples	45,416
Training set	36,333 (80%)
Independent test set	9,083 (20%)
Data-partition strategy	Grouped stratified random split
Random seed	42
Grouping criterion	Identical S1–S8 response vectors
Cross-validation	5-fold CV on training set only
Identical S1–S8 vectors shared by Train/Test	0
Mean 5-fold Accuracy	0.999945
Independent-test Accuracy	0.9969
Independent-test Precision	0.9970
Independent-test Recall	0.9969
Independent-test F1-score	0.9969

Table S4. Hyperparameter settings of the classification and prediction models.

Parameter	1D-CNN Classification Model	Proposed Prediction Model
Network architecture	2 Conv1D layers	Bi-GRU + Attention + MTL
Kernel size	2	N/A
Hidden units / filters	64, 128 filters	128 GRU units
Learning rate	0.001	0.001
Window length	N/A	12
Optimizer	Adam	Adam
Batch size	32	32
Loss function	Categorical cross-entropy	Huber loss
Maximum epochs	200	1000
